# A randomized controlled trial to investigate the impact of a low glycemic index (GI) diet on body mass index in obese adolescents

**DOI:** 10.1186/1471-2458-14-180

**Published:** 2014-02-19

**Authors:** Alice PS Kong, Kai Chow Choi, Ruth SM Chan, Kris Lok, Risa Ozaki, Albert M Li, Chung Shun Ho, Michael HM Chan, Mandy Sea, C Jeyakumar Henry, Juliana CN Chan, Jean Woo

**Affiliations:** 1 Department of Medicine and Therapeutics, The Chinese University of Hong Kong, Hong Kong, SAR, China; 2 Nethersole School of Nursing, The Chinese University of Hong Kong, Hong Kong SAR, Shatin, N.T., Hong Kong, SAR, China; 3 Department of Paediatrics, The Chinese University of Hong Kong, Hong Kong, SAR, China; 4 Department of Chemical Pathology, The Chinese University of Hong Kong, Hong Kong, SAR, China; 5 Li Ka Shing Institute of Health Sciences, The Chinese University of Hong Kong, Hong Kong, SAR, China; 6 Director, Clinical Nutrition Research Centre, Oxford, Singapore

**Keywords:** Glycemic index, Obesity, Adolescents, Chinese

## Abstract

**Background:**

The role of a low glycemic index (GI) diet in the management of adolescent obesity remains controversial. In this study, we aim to evaluate the impact of low GI diet versus a conventional Chinese diet on the body mass index (BMI) and other obesity indices of obese adolescents.

**Methods:**

Obese adolescents aged 15–18 years were identified from population-recruited, territory-wide surveys. Obesity was defined as BMI ≥95th percentile of Hong Kong local age- and sex-specific references. Eligible subjects were randomized to either an intervention with low GI diet (consisting of 45-50% carbohydrate, 30-35% fat and 15-20% protein) or conventional Chinese diet as control (consisting of 55-60% carbohydrate, 25-30% fat and 10-15% protein). We used random intercept mixed effects model to compare the differential changes across the time points from baseline to month 6 between the 2 groups.

**Results:**

104 obese adolescents were recruited (52 in low GI group and 52 in control group; 43.3% boys). Mean age was 16.7 ± 1.0 years and 16.8 ±1.0 years in low GI and control group respectively. 58.7% subjects completed the study at 6 months (65.4% in low GI group and 51.9% in control group). After adjustment for age and sex, subjects in the low GI group had a significantly greater reduction in obesity indices including BMI, body weight and waist circumference (WC) compared to subjects in the control group (all p <0.05). After further adjustment for physical activity levels, WC was found to be significantly lower in the low GI group compared to the conventional group (p = 0.018).

**Conclusion:**

Low GI diet in the context of a comprehensive lifestyle modification program may be an alternative to conventional diet in the management of obese adolescents.

**Trial registration number:**

ClinicalTrials.gov Ref. No: NCT01278563

## Background

Obesity is a prevalent condition in both the adult and in the adolescent population. The prevalence and severity of obesity in both children and in adolescents are increasing worldwide [1,2] and there is substantial tracking of childhood overweight/obesity into adulthood [3]. It is evident that childhood obesity is not a benign condition and is associated with clustering of cardiovascular risk factors and co-morbidities [4-6]. Most worrying is the lack of effective therapies in the management of childhood obesity. Conventional weight management programs have focused on restricting energy consumption and limiting fat and/or carbohydrate intake. However, the effectiveness of these conventional dietary approaches for children and adolescents are only modest and inconclusive due to the small number of trials and their short-term nature [7,8]. There is growing evidence showing that when access to palatable food is restricted, this will promote the tendency to overeat when these food become freely available [9]. The emphasis of low fat diet may theoretically increase glycemic index (GI), which has been implicated in the pathogenesis of diabetes and cardiovascular diseases [10,11].

Emerging evidence suggests that low GI diet may be more efficacious than an energy-restricted, low fat diet in reducing body weight [12-14]. Against this background, we conducted this randomized controlled trial aiming to study the changes in obesity indices in obese adolescents randomized to low GI diet versus a conventional Chinese diet as control.

## Methods

### Study population and assessments

The study commenced in February 2010 and each subject was followed up for 18 months consisting of a 12-month interventional period followed by a 6-month observational period. This is the interim analysis of the data at 6-months for this ongoing study. This study was approved by the Clinical Research Ethics Committee of the Chinese University of Hong Kong. Written informed consent was obtained from all participants as well as from their parents’ or guardians’ prior to recruitment into the study.

Obese adolescents were identified from territory-wide, population-recruited surveys [2,4,15]. All participants from the surveys were healthy volunteers with no major medical illnesses and not on any chronic medications. Obesity was defined as body mass index (BMI) ≥95th percentile of local age- and sex-specific references [16]. Eligible subjects were randomized to 2 groups: low GI diet versus conventional Chinese diet. Randomization were carried out using computer-generated random numbers, sealed in opaque envelopes and in blocks of 6 further stratified by gender. Treatment assignment was done by an independent personnel who opened the envelopes with consecutive numbers for the allocation of treatment of eligible subjects. All participants underwent a comprehensive assessment including anthropometric measurements, clinical examinations by endocrinologists and questionnaires documenting food frequency and 3-day diet record using locally validated questionnaires [17], as well as physical activity levels using a locally validated Chinese version of International Physical Activity Questionnaires (IPAQ) [18]. The scoring protocol for physical activity level was downloaded from IPAQ official website (http://www.ipaq.ki.se/scoring.htm). Resting energy expenditure was measured by indirect calorimetry using MedGem® (Microlife Medical Home Solutions Inc., United States). Body fat percentage was assessed by bioimpedence (Tanita physician digital scale, model number TBF 410, Tanita Corp., Tokyo, Japan).

After an overnight fast of 8–10 hours, blood samples were collected for measurement of fasting glucose and lipid profile including total cholesterol (TC), triglyceride (TG) and high-density lipoprotein cholesterol (HDL-C) levels. Low-density lipoprotein cholesterol (LDL-C) level was calculated using the Friedewald’s formula for TG < 4.5 mmol/l [19]. An oral glucose tolerance test (OGTT) was performed with the administration of 1.75 g of glucose per kilogram of body weight with maximal dose of 75 g. Diabetes was diagnosed if fasting plasma glucose ≥ 7.0 mmol/L and/or 2 hour plasma glucose ≥ 11.1 mmol/L [20]. Impaired fasting glucose (IFG) was diagnosed if fasting plasma glucose was 5.6-6.9 mmol/L and impaired glucose tolerance (IGT) was diagnosed if 2 hour plasma glucose was 7.8-11.0 mmol/L [20]. Laboratory assessments were all repeated at 6 months.

### Interventions

Subjects allocated to the low GI group were counselled by a dietitian at week 0, 2, 4, 6, 8 and thereafter at 8-week intervals, for a total of 7 sessions during the 6-month intervention period. Each session lasted approximately 30 minutes on an individual basis. Parents of the subject were encouraged to join the counselling sessions. The intervention was based on a strategy of increasing energy expenditure and reducing caloric intake using lifestyle behavioral change to achieve long-lasting impact. The dietitian completed a tracking form and progress note after each counselling session to document patterns of dietary intake. Three-day diet record which included two weekdays and a weekend day as reported by the participants prior to the consultation visits, were reviewed by the dietitian with appropriate advice given. Physical activity levels were measured by self-administered, validated questionnaires [18,21].

In the first session (about 1 hour) (week 0), the dietitian carried out a complete behavioral assessment covering the participant’s current eating and lifestyle patterns, nutritional knowledge, and feelings about lifestyle changes. The dietitian discussed with the participants the expected duration and specific dietary and lifestyle advice required to achieve a desirable weight status (<85th percentile of the local age- and sex-specific reference) [16]. The dietitian prescribed an individualized menu plan with 20% caloric restriction based on his/her current diet. The dietitian would also consider the daily nutritional requirement of the participant based on the recommendations of the American Dietetic Association [22]. A balanced diet with an emphasis on fruit and vegetables, and low-fat, low-GI and low-calorific products in appropriate portions was encouraged. Low caloric products are those equal to or less than 100 kcal per serving, and the fat and sugar content should also be equal to or less than 5 gm and 30% of the total calorie respectively. Practical tips were given based on behavioral principles including goal setting, knowledge acquisition, problem solving, feedback and reinforcement. Participants and their families were educated on the benefits of a low GI diet. Each participant received two booklets, one for food portion size exchange and tips for eating out, and another listing the low GI food options and meal plans (GI <55) [23]. Consumption of low GI food, use of healthy fat, and avoidance of high GI food based on the low GI pyramid [24] were emphasized. Participants were encouraged to have at least one low-GI food per meal. Participants and their families were taught how to read food labels to ensure balanced intake. The targeted proportion of energy from carbohydrate and fat were 45-50% and 30-35%, respectively, with the rest coming from protein.

In the follow-up sessions (lasting for about 20 minutes), the dietitian reviewed the participant’s compliance to diet as well as his/her progress by reviewing the dietary record to ensure adequate nutritional calories in the diet and provide appropriate recommendations on the dietary plan. Besides reviewing the participant’s compliance to the diet, the dietitian encouraged the participants to share any personal and environmental barriers to lifestyle change. The dietitian also assessed the participant’s feelings towards the dietary advice and to the progress made. Ongoing support and encouragement was provided to the participants, with target goals re-defined based on the participant’s feelings and progress, and the participant’s efforts and achievements was acknowledged to enhance their self-efficacy. Participants were encouraged to perform aerobic exercise ≥3 days of 30 minutes a week. Motivational telephone calls (10–15 minutes) were made 5 times during the 6-month period to remind them to adhere to the assigned diet and to address their queries on food choices.

In order to standardize the dietary advice in the control group, the conventional Chinese diet were based on the standard food pyramid promoted by the Hong Kong Department of Health with advice on daily proportion of carbohydrate, fat and protein without information about low GI diet [25]. The targeted proportion of energy from carbohydrate and fat were 55-60% and 25-30%, respectively, with the rest coming from protein. The conventional Chinese diet was presumed to be a high GI diet (≥70) based on several local studies showing that refined grains, especially Jasmine rice from Thailand with a GI value of 109 were commonly consumed among Hong Kong Chinese children and their adult counterparts [26]. Control group was counselled by a research nurse during the intervention period. Dietary advice was based on the dietary recommendation of the standard food pyramid, with emphasis on reducing energy intake by limiting dietary fat intake and high caloric foods [25].

The contact time, both individual counseling and telephone reinforcement, and the dietary assessment methods were the same for participants randomized to the control group and to the low GI group. Subject participation was evaluated on the basis of session attendance, and adherence to dietary advice was assessed by means of the 3-day diet record.

### Dietary assessment

Daily nutritional intake and food consumption at baseline and 6 months for participants of both groups were assessed using a three-day diet record. Participants were asked to provide detailed descriptions of foods and beverages consumed. Common household measures such as cups, bowls, teaspoons and tablespoons, etc., as well as a food photo album consisting of common food items were used to quantify food intake. Daily nutritional intake and food consumption at baseline and 6 months were calculated by the nutrition analysis software *Food Processor Nutrition analysis and Fitness software version 8.0* (ESHA Research, Salem, USA) including local foods selected from food composition tables from China and Hong Kong [27,28]. Dietary values of GL and glycemic load (GL) of the participants were estimated using a Food Frequency Questionnaire (FFQ) at baseline and 6 months [17]. Each participant was asked to complete the questionnaire – the food item, the size of each portion, the number of times of consumption each day and each week, using the past six months prior to the interview as a reference period. Portion size was explained to participants using a catalogue of pictures of individual food portions. The GI of each individual food based on the glucose reference in the FFQ were assigned according to published values obtained from the international table of GI and GL values of foods [29], the China Food Composition Table [27], one Hong Kong paper [30], as well as from database from Sydney [31]. When a published value was not available, the composition of the food was systematically evaluated to impute a GI value. The dietary GI for each participant was calculated by summing the products of the percentage contribution of each individual food to daily available carbohydrate intake multiplied by the food’s GI value. Available carbohydrate was calculated as total carbohydrate minus dietary fiber [32]. The dietary GL was also calculated by multiplying the dietary GI by the total amount of daily available carbohydrate intake (divided by 100).

### Laboratory assays

Plasma glucose (hexokinase method), TC (enzymatic method), TG (enzymatic method without glycerol blanking) and HDL-C (direct method using PEG-modified enzymes and dextran sulfate) were measured on a Roche Modular Analytics system (Roche Diagnostics GmbH, Mannheim, Germany) using standard reagent kits supplied by the manufacturer of the analyzer. The precision performance of these assays was within the manufacturer’s specifications.

### Statistical analyses

Data were summarized and presented using appropriate statistics; mean (standard deviation), median (inter-quartile range) and frequency (percentage) for normal-like, skewed and categorical variables respectively. Visceral fat was log-transformed to correct for its skewness before being entered for statistical analysis. Baseline characteristics between the two arms of subjects were compared using Student’s t test or chi-square test, as appropriate. All the outcome variables were analysed on the basis of the intention-to-treat (ITT) principle. Random intercept mixed effects model was used to compare the differential changes on various outcome measures of obesity indices and cardiometabolic risk factors (BMI, obesity indices and cardiometabolic risk factors were primary, secondary and tertiary outcome measures respectively) across the time points at month 0 (baseline) and month 6 between the two arms with adjustment for age and sex. In particular, the interaction terms group*time was included in the mixed effects model to assess the difference in change of each of the outcomes across month 0 and month 6 between the two arms. Mixed effects model can account for intra-correlated repeated measures data and accommodate missing data due to incomplete visits or subjects who have dropped-out, provided the data are missing at random [33], and thus is particularly suitable for intention-to-treat analysis without the need of imputation for missing data. The mixed effects models were analysed using the PROC MIXED (SAS Institute, Cary, NC, release 9.3). Other statistical analyses were performed using SPSS 18.0 (SPSS Inc., Chicago, IL). All statistical tests involved were two-sided and a p-value <0.05 was considered statistically significant.

## Results

104 obese adolescents were recruited in this study (52 in the low GI group and 52 in the control group; 43.3% boys; mean age 16.8 ± 1.0 years; mean BMI 30.9 ± 3.9 kg/m^2^). Table 1 shows the baseline clinical and biochemical characteristics of the study cohort. Obesity indices between adolescents in the control and low GI groups were similar except adolescents in control group were less centrally obese and tended to have lower body fat compared to the low GI group [waist circumference and body fat percentage of the control and low GI group were 92.3 ± 9.8 and 96.5 ± 10.9 cm (p = 0.043), and 36.8 ± 8.3 and 39.8 ± 10.9% (p = 0.051) respectively]. All other clinical and biochemical characteristics of the study subjects in both groups were comparable (all p > 0.05). None of them had diabetes or impaired fasting glucose at baseline, but 14% of them had impaired glucose tolerance (Table 1). 58.7% subjects completed 6 month study (65.4% in low GI group and 51.9% in control group).

**Table 1:** Characteristics of the participants at the baseline (n = 104)

**Characteristics**	**Control (n = 52)**	**Low GI (n = 52)**	**p**^**#**^
Sex^ψ^			
Female	28 (53.8%)	31 (59.6%)	0.553
Male	24 (46.2%)	21 (40.4%)	
Age (years)	16.7 (1.0)	16.8 (1.0)	0.797
Body weight (kg)	82.9 (14.9)	87.6 (13.0)	0.090
Body height (m)	1.65 (0.09)	1.66 (0.08)	0.444
Body mass index (kg/m^2^)	30.2 (3.5)	31.6 (4.2)	0.068
Waist circumference (cm)	92.3 (9.8)	96.5 (10.9)	0.043
Systolic blood pressure (mmHg)	121.6 (10.2)	123.1 (9.7)	0.462
Diastolic blood pressure (mmHg)	72.4 (8.7)	74.0 (8.0)	0.326
Body fat (%)	36.8 (8.3)	39.8 (7.3)	0.051
Trunk fat (%)	40.5 (7.2)	42.9 (6.1)	0.071
Visceral fat (%)^†^	12.5 (9.0 – 19.0)	14.2 (10.8 – 19.8)	0.367
Total cholesterol (mmol/L)	4.4 (0.7)	4.2 (0.7)	0.303
HDL-cholesterol (mmol/L)	1.3 (0.3)	1.2 (0.3)	0.674
LDL-cholesterol (mmol/L)	2.6 (0.7)	2.5 (0.6)	0.580
Non HDL-cholesterol (mmol/L)	3.1 (0.7)	3.0 (0.7)	0.416
Triglyceride (mmol/L)	1.1 (0.4)	1.0 (0.4)	0.236
Urine ACR (mg/mmol)^†^	0.45 (0.26 – 0.84)	0.56 (0.33 – 1.32)	0.069
Fasting plasma glucose (mmol/L)	4.6 (0.3)	4.7 (0.3)	0.071
Two-hour FPG (mmol/L)	6.0 (1.3)	6.1 (1.2)	0.642
AUC glucose (10^2^ mmol/L * min)	8.1 (1.3)	8.5 (1.3)	0.176
Diabetes status^ψ^			
Normal	44 (84.6%)	46 (88.5%)	0.566
Impaired glucose tolerance	8 (15.4%)	6 (11.5%)	

Variables marked with ^ψ^ are presented as frequency (%), ^†^ are presented as median (interquartile range), all others are presented as mean (standard deviation). HDL: high density lipoprotein cholesterol; LDL: low density lipoprotein cholesterol; ACR: albumin-to-creatinine ratio; AUC glucose: area under the curve of glucose levels across 2 hours of a standard oral glucose tolerance test.

^#^Categorical and continuous variables were compared between the two groups using Pearson chi-square test and t-test respectively, those marked with ^†^ were log-transformed before being tested by t-test.

Table 2 shows the daily dietary intake of macronutrients and fiber, resting energy expenditure and physical activity level of the study subjects at baseline and 6 months of the study. There was no significant difference in daily nutrient intakes, resting energy expenditure and physical activity level between the two groups at baseline. At 6 months, adolescents in the low GI group had significantly lower total daily energy intake, less fat intake, and more protein and fiber intake in their diet (all p < 0.05) compared to adolescents in the control group (Table 2). However, we could not find a significant difference in GI and glycemic load (GL) between the two groups from baseline to month 6 (Table 2).

**Table 2:** Daily dietary intake of macronutrients and fiber, resting energy expenditure and physical activity level across month 0 and month 6 of the study subjects in both arms [the control and the low glycemic index (GI) groups]

	**Month 0**			**Month 6**		
	**Control (n = 52)**	**Low GI (n = 52)**	**p**^**#**^	**Control (n = 27)**	**Low GI (n = 34)**	**p**^**#**^
**Nutrient**						
Energy (kcal)						
Observed	2102.9 (544.3)	1955.2 (583.4)	0.185	1981.6 (653.7)	1565.0 (545.1)	0.001
Expected	–	–		–	–	
Carbohydrate (% of energy)						
Observed	47.9 (5.6)	47.2 (8.2)	0.598	49.5 (6.6)	50.8 (8.1)	0.384
Expected	–	–		55–60%	45–50%	
Fat (% of energy)						
Observed	35.6 (4.9)	35.6 (6.8)	0.975	34.4 (5.9)	31.2 (6.7)	0.012
Expected	–	–		25–30%	30–35%	
Protein (% of energy)						
Observed	16.5 (2.4)	17.1 (3.4)	0.253	15.9 (2.7)	17.9 (3.8)	0.002
Expected	–	–		10–20%	15–25%	
Fiber (g/1000 kcal)						
Observed	4.9 (1.9)	5.2 (3.2)	0.538	5.3 (2.5)	6.5 (3.3)	0.041
Expected	–	–		–	–	
GI						
Observed	76.1 (10.8)	74.4 (10.5)	0.417	76.8 (10.2)	74.4 (8.7)	0.199
Expected	–	–		–	–	
GL, per 1000 kcal						
Observed	110.5 (46.3)	115.6 (50.5)	0.591	106.3 (42.7)	117.7 (42.5)	0.175
Expected	–	–		–	–	
**Resting energy expenditure (kcal/kg)**	22.6 (4.4)	21.4 (3.1)	0.130	23.7 (4.0)	22.3 (4.1)	0.174
	*(n = 35)	*(n = 35)		*(n = 18)	*(n = 28)	
**Physical activity level (MET-minutes/week)**^**†**^	1626 (594 – 3657)	3492 (1386 – 4506)	0.143	2266 (960 – 4318)	1453 (740 – 2780)	0.235

Variables marked with ^†^ are presented as median (interquartile range), all others are presented as mean (standard deviation).

GL: glycemic load.

^#^t-test between the control and low GI groups; those variables marked with ^†^ were log-transformed before being compared by t-test.

*The number of valid responses to the International Physical Activity Questionnaire (IPAQ), which was used for assessing the average physical activity level of the participants in the past seven days.

Table 3 shows the obesity indices and other cardiometabolic risk factors including blood pressure, lipid profile, indices of glycemia and albuminuria of the subjects at baseline and 6-month in both groups. After adjustment for age and sex, subjects in the low GI group had significant reduction in obesity indices including BMI, body weight and waist circumference compared to subjects in the control group (all p < 0.05) (Table 4). After adjustment for age, sex and physical activity, waist circumference remained significantly lower in the low GI group compared to that in the conventional group after 6 months of intervention (p = 0.018) (Table 5). We did not find any significant differences in body fat percentage (total, truncal and visceral fat percentage) as assessed by bioimpedence at baseline and across month 6 of the study (Tables 4 and 5). We also did not find any significant changes across baseline and month 6 for other cardiometabolic risk factors including blood pressure, LDL-cholesterol, HDL-cholesterol, triglyceride, fasting plasma glucose, 2-hour plasma glucose and glucose changes during OGTT (as measured by area under curve of OGTT) (data not shown).

**Table 3:** Obesity indices and other cardiometabolic risk factors across month 0 and month 6 of study subjects in both arms (control and low glycemic index, GI, groups)

	**Month 0**		**Month 6**	
	**Control (n = 52)**	**Low GI (n = 52)**	**Control (n = 27)**	**Low GI (n = 34)**
**Obesity indices**				
Body weight (kg)	82.9 (14.9)	87.6 (13.0)	83.0 (13.1)	84.3 (14.4)
Body mass index (kg/m^2^)	30.2 (3.5)	31.6 (4.2)	30.0 (2.9)	30.4 (4.0)
Waist circumference (cm)	92.3 (9.8)	96.5 (10.9)	90.5 (10.2)	91.2 (10.5)
Body fat (%)	36.8 (8.3)	39.8 (7.3)	35.9 (7.4)	38.5 (8.9)
Trunk fat (%)	40.5 (7.2)	42.9 (6.1)	39.0 (7.1)	41.6 (6.9)
Visceral fat (%)^†^	12.5 (9.0 – 19.0)	14.2 (10.8 – 19.8)	13.5 (7.5 – 20.5)	12.0 (10.0 – 19.0)
**Other cardiometabolic risk factors**				
Systolic blood pressure (mmHg)	121.6 (10.2)	123.1 (9.7)	120.7 (10.6)	123.0 (10.0)
Diastolic blood pressure (mmHg)	72.4 (8.7)	74.0 (8.0)	72.4 (9.8)	74.3 (7.0)
Total cholesterol (mmol/L)	4.4 (0.7)	4.2 (0.7)	4.2 (0.8)	4.0 (0.8)
HDL-cholesterol (mmol/L)	1.3 (0.3)	1.2 (0.3)	1.2 (0.3)	1.2 (0.2)
LDL-cholesterol (mmol/L)	2.6 (0.7)	2.5 (0.6)	2.4 (0.7)	2.3 (0.7)
Non HDL-cholesterol (mmol/L)	3.1 (0.7)	3.0 (0.7)	3.0 (0.7)	2.8 (0.8)
Triglyceride (mmol/L)	1.1 (0.4)	1.0 (0.4)	1.3 (0.5)	1.1 (0.5)
Urine ACR (mg/mmol)^†^	0.45 (0.26 – 0.84)	0.56 (0.33 – 1.32)	0.79 (0.32 – 1.44)	0.55 (0.30 – 0.99)
Fasting plasma glucose (mmol/L)	4.6 (0.3)	4.7 (0.3)	4.7 (0.5)	4.7 (0.4)
Two-hour FPG (mmol/L)	6.0 (1.3)	6.1 (1.2)	6.0 (1.5)	6.0 (2.0)
AUC glucose (10^2^ mmol/L * min)	8.1 (1.3)	8.5 (1.3)	8.4 (1.6)	8.6 (2.1)

Variables marked with ^†^ are presented as median (interquartile range), all others are presented as mean (standard deviation).

HDL-cholesterol: high density lipoprotein cholesterol; LDL-cholesterol: low density lipoprotein cholesterol; ACR: albumin-to-creatinine ratio; AUC glucose: area under the curve of glucose levels across 2 hours of a standard oral glucose tolerance test.

**Table 4:** Comparison of the cardiometabolic risk outcome variables across 0 and 6 months between the study subjects in the control and low glycemic index, GI, group after adjustment for age and sex

	**Regression coefficients of the mixed effects models**					
	**Group**		**Time**		Group*Time	
**Obesity indices**	**B (95% CI)**	**p**	**B (95% CI)**	**p**	**B (95% CI)**	**p**
Body weight (kg)	5.30 (0.53, 10.08)	0.029	-0.03 (-1.25, 1.19)	0.968	-2.56 (-4.75, -0.38)	0.022
Body mass index (kg/m^2^)	1.41 (-0.05, 2.86)	0.058	-0.17 (-0.59, 0.24)	0.414	-0.98 (-1.81, -0.14)	0.023
Waist circumference (cm)	4.55 (0.95, 8.16)	0.013	-1.87 (-2.95, -0.79)	0.001	-3.57 (-5.70, -1.44)	0.001
Body Fat (%)	2.52 (0.09, 4.95)	0.042	-0.09 (-1.28, 1.10)	0.882	-1.49 (-3.98, 1.00)	0.241
Trunk fat (%)	2.16 (-0.20, 4.51)	0.073	-0.66 (-2.12, 0.81)	0.379	-0.81 (-2.75, 1.13)	0.411
Visceral fat (%)^†^	0.11 (-0.03, 0.24)	0.122	-0.03 (-0.11, 0.06)	0.553	-0.04 (-0.15, 0.08)	0.547

Those outcomes marked with ^†^ were log-transformed before being subjected to analysis.

Only the model estimates of the regression coefficient (B) of the dummy variables for the group [Group: 0 = Control (reference); 1 = Low GI], time (Month 6 and month 0 as reference), time and group interaction terms (Group*Time) were shown for the mixed effects models with adjustment for age and sex.

**Table 5:** Comparison of the cardiometabolic risk outcome variables across 0 and 6 months between the study subjects in the control and low glycemic index, GI, group after adjustment for age, sex and physical activity level

	**Regression coefficients of the mixed effects models**					
	**Group**		**Time**		Group*Time	
**Obesity indices**	**B (95% CI)**	**p**	**B (95% CI)**	**p**	**B (95% CI)**	**p**
Body weight (kg)	5.17 (0.18, 10.17)	0.043	-0.21 (-2.19, 1.77)	0.836	-1.65 (-4.67, 1.36)	0.283
Body mass index (kg/m^2^)	1.15 (-0.29, 2.58)	0.117	-0.12 (-0.80, 0.56)	0.721	-0.82 (-1.96, 0.33)	0.161
Waist circumference (cm)	4.17 (0.28, 8.07)	0.036	-2.59 (-4.09, -1.10)	0.001	-3.47 (-6.36, -0.59)	0.018
Body Fat (%)	2.46 (-0.46, 5.38)	0.099	1.21 (-0.94, 3.36)	0.269	-2.46 (-6.18, 1.27)	0.196
Trunk fat (%)	2.83 (0.16, 5.51)	0.038	-0.96 (-2.51, 0.60)	0.228	-0.78 (-3.33, 1.77)	0.547
Visceral fat (%)^†^	0.15 (0.02, 0.28)	0.024	-0.07 (-0.14, 0.01)	0.068	0.00 (-0.16, 0.15)	0.960

Those outcomes marked with ^†^ were log-transformed before being subjected to analysis.

Only the model estimates of the regression coefficient (B) of the dummy variables for the group [Group: 0 = Control (reference); 1 = Low GI], time (Month 6 and month 0 as reference), time and group interaction terms (Group*Time) were shown for the mixed effects models with adjustment for age, sex and physical activity level.

## Discussion

There is a paucity of data in dietary intervention studies examining the impact of low GI diet in obese adolescents. To date, there has only been one published study from Europe (the Diogenes Project) of a 6-month intervention [34] which had a total of 5 intervention arms (low protein/low GI, low protein/high GI, high protein/low GI, high protein/high GI and control diet). It concluded that neither GI nor protein had an isolated effect on body composition. Nonetheless, the low protein and high GI combination increased body fat while the high protein and low GI diet was protective against obesity in children. Our present study is novel in being the largest randomized controlled trial with a reasonable duration of intervention, having low GI diet as the only intervention arm [35]. Moreover, all the participants were of Chinese ethnicity and our study included the measurements of resting energy expenditure and physical activity levels, which were not included in the work reported by Papadaki and co-workers [34]. We found that obese adolescents had significantly greater reduction in obesity indices after 6 months of intervention with low GI dietary counseling compared to those in the control group. The obese adolescents in the low GI group also had less total energy intake and a healthier diet with greater daily dietary fiber and protein intake, and less fat intake. However, despite the fact that the low GI group had greater daily dietary intake of fiber and less carbohydrate, we could not show a significant difference in GI and GL between the two groups from baseline to month 6. There are a number of possible explanations for this lack of differences of GI and GL between the two groups at baseline and after 6 months. First, it might be possible that the study subjects randomized to low GI diet did not comply satisfactorily to the prescribed diet. From our observation, most participants in the intervention group did not include at least one low GI food per meal. Only some of them were able to replace high GI food items with low GI ones, such as white rice with brown or red rice, white bread with whole wheat bread, or classic coke with diet coke. Second, the energy and macronutrients, including energy, carbohydrate, fat and protein were estimated by 3-day record which was considered more accurate in capturing current energy and macronutrient intakes. However, the GI and GL values from diet were estimated by a food frequency questionnaire (FFQ). FFQ had only been validated for quantifying common nutrient intakes but had not been validated for estimating dietary GI and GL values. Third, many Chinese food do not have GI values and were actually difficult to provide GI values, such as Dim Sum. The GI values from diets of the study subjects in this present trial were estimated mainly from the GI values of international published data which might account for some bias in the accurate estimation of GI values of Chinese food.

Conventional weight management programs have focused on restricting energy consumption and limiting fat and/or carbohydrate intake. However, the effectiveness of these conventional dietary approaches for children and adolescents are only modest and inconclusive due to the limited number of trials, small number of subjects in the trials and their short-term nature [7,8,35]. Besides, high attrition rates, lack of assessment of adherence to treatment protocols and unequal intensity of intervention across intervention arms are important limitations for many dietary intervention trials and possibly account for the inconsistent results reported among different studies [24,36,37]. There is indeed a stark need to search for effective nutritional management in obese adolescents.

Increasing evidence from epidemiological studies from the East and West show that dietary GI may play a protective role in reducing the risks of chronic diseases such as obesity, type 2 diabetes and coronary heart diseases [10,11,35]. Although the exact mechanisms underlying the beneficial effect of low GI are not fully understood, it is plausible that low GI food work through reducing satiety and attenuating pancreatic insulin responses post-prandially. In energy homeostasis, multiple hormones with metabolic and haemodynamic effects are involved in the brain-gut-pancreas-liver axis [35]. Habitual consumption of high GI diet will initiate a sequence of metabolic and neurohormonal events that can stimulate hunger, promote fat deposition, increase insulin secretion, thereby putting the pancreatic beta cells under chronic stress resulting in early onset of type 2 diabetes [35,38], especially in the presence of other risk factors such as genetic variants [39]. In rat models, high GI food have been shown to increase post-prandial rise in plasma glucose and insulin levels, increase plasma triglyceride concentrations, decrease adiponectin levels, increase body fat and decrease lean body mass [38]. In human studies, intake of low GI food has also been shown to increase lean body mass, cause greater weight loss and less inflammatory activation compared to high GI diet [38,40]. In overweight or obese adults, consumption of low GI food is associated with increased resting energy expenditure, decreased plasma free fatty acids levels, reduced fat oxidation, improved lipid profile and increased satiety [38,40,41].

An interesting observation in this study is that 0 out of 104 obese adolescents had abnormal fasting plasma glucose levels at baseline. Nonetheless, 14% of the participants had impaired glucose tolerance which was detected only by OGTT. First phase insulin secretion and post-prandial hyperglycemia are early signs of diabetes in people from Asia. This highlights the importance of performing OGTT in at-risk individuals, such as those with obesity and of Asian ethnicity.

Our present study has the strength of close monitoring to treatment protocols adherence by regular telephone reminders and equal intervention across the 2 arms. There were equal contact times, counseling and telephone reinforcement, as well as the dietary assessment methods for the participants in both the control group and the low GI group. We had measurements of energy expenditure by using indirect calorimetry and IPAQ which were lacking in the European trial reported by Papadaki [34]. There are several limitations which need to be addressed in this study. First, we used dietary questionnaires to assess macronutrients of the study subjects before, during and after intervention. Due to the fact that self-reported dietary intake measures is a common research limitation, there has been increasing use of dietary biomarkers for research in the field of nutrition [42]. Of note, this was not only a low-GI intervention, since there were other intervention components as well (e.g. weight loss). Compared to the DiOGenes study which involved an ad libitum energy intake [34], the present study was a weight loss study promoting a low-GI diet. Therefore, it might be difficult to distinguish whether the effects observed were due to the low-GI advice provided or the reduction in energy intake that was promoted. Second, our study had high attrition rate with only 59% subjects completing the study at 6 months despite our research team’s efforts in giving telephone reminders and reinforcement, which was indeed comparable to the attrition rate reported by the only published 6-month dietary intervention including low GI diet in obese adolescents [34]. The high drop-out rate is a common phenomenon in dietary interventions and particularly a challenge for dietary trials in adolescents who are free of any symptoms and with multiple competing commitments such as school work and social activities. For the dietary intervention done by the same group of researchers (Diet, Obesity and Genes, Diogenes, Project), 58.1% adolescents completed 6 months dietary intervention [34] while 71% adults completed the 6-month trial [43]. Third, our study captures outcome measures at 6 months which may be too short to detect any significant change in lipid profile and glucose intolerance. Moreover, the planned sample size for this ongoing study would be 140 subjects per study arm based on the meta-analysis of randomized controlled trials including both adults and adolescents studies that assessed the effects of low GI diet versus high GI or other diets for change in body mass index [40], the data may be under-powered to detect changes in cardiometabolic risk factors. These factors may account for our negative findings of any differences in glycemia and lipid profile between participants in the control and the low GI group. Longer duration of intervention would provide more information in this regard. Fourth, we did not document the data on the compliance to the dietary requirements of low GI diet or American Diabetes Association conventional diet. Nonetheless, in each counseling session, the dietitian did review the dietary records and provided recommendations accordingly.

## Conclusion

In conclusion, this 6-month dietary intervention trial results in significant reduction in obesity indices in obese adolescents when compared to conventional diet. The 6-month intervention resulted in decreased calorie intake and a healthier dietary composition in terms of increased fiber intake and reduced fat intake in obese adolescents. Our results and others suggest that apart from its quantity, the quality of carbohydrate content in a meal may be an essential element in obesity management with health implications [44]. A low GI diet may be an alternative to conventional approach in dietary management of obese adolescents and the importance of a comprehensive lifestyle modification program cannot be over-emphasized in obesity management. A longer duration randomized controlled dietary trial is ongoing and will provide more information regarding the longer term effects of low GI diet on other metabolic profiles.

## Competing interest

All authors had no conflicts of interest to declare relating to this manuscript.

## Authors’ contributions

APSK, KCC and RSMC have full access to all of the data in the study and take responsibility for the integrity of the data and the accuracy of the data analysis. Study concept and design: APSK, CJKH, JCNC and JW Acquisition of data: KL and RSMC. Analysis and interpretation of data: APSK, KCC and RSMC. Drafting of the manuscript: APSK, KCC, RSMC and JCNC. Critical revision of the manuscript: RO, AML, CJKH, JW. Statistical analysis: KCC and RSMC. Obtained funding: APSK, JCNC. Supervision of the laboratory procedures: CSH and MHMC. Supervision of the intervention: RSMC and MS. All authors read and approved the final manuscript.

## Pre-publication history

The pre-publication history for this paper can be accessed here:

http://www.biomedcentral.com/1471-2458/14/180/prepub
